# E-cigarette use (vaping) and cigarette smoking are associated with reduced markers of ovarian reserve: a cross-sectional study of 16,087 women

**DOI:** 10.1186/s12905-026-04620-x

**Published:** 2026-06-23

**Authors:** Natalie Getreu, Esther Wainwright, Lauren Crawford, Silvia Nedelcu, Bríd Ní Dhonnabháin, Sofia Rodrigues Vaz, Tharni Vasavan, Helen C. O’Neill

**Affiliations:** 1 Research & Development Department Hertility Health Ltd., London, UK; 2 Institute for Women’s Health, University College London (UCL), London, UK; 3 Aberdeen Reproductive Medicine Unit, NHS Grampian, Aberdeen, UK; 4 Institute of Applied Health Sciences, University of Aberdeen, Aberdeen, UK

**Keywords:** E-cigarette use, Vaping, Cigarette smoking, Ovarian reserve, Anti-Müllerian hormone, Follicle-stimulating hormone, Reproductive health, Cross-sectional study

## Abstract

**Background:**

E-cigarette use (vaping) is rapidly increasing among women of reproductive age, with an estimated one in five women aged 15–45 now using e-cigarettes in England. Despite being promoted as a safer alternative to cigarettes, the reproductive health consequences of e-cigarettes remain poorly understood, despite products being on the market for 2 decades. This study aimed to investigate the association between e-cigarette use, cigarette smoking, and two hormonal markers of ovarian reserve in women of reproductive age.

**Methods:**

A cross-sectional analysis was conducted using data from 16,087 women aged 18-45 who accessed a private reproductive hormone testing service between 11/2022 and 02/2025. Women with pre-existing reproductive health conditions (including PMOS, endometriosis, POI, and hypothalamic amenorrhoea) and those using hormonal contraception were excluded, ensuring a reproductively healthy population for analysis. Self-reported vaping and smoking were primary exposures, captured as never, former, occasional, or current use. Serum AMH and FSH were measured via standardised immunoassay from capillary blood samples. Multiple linear regression with log-transformed outcomes was performed, adjusting for age, BMI, and ethnicity. Vaping and smoking were modelled simultaneously to account for overlap between the two behaviours.

**Results:**

Current vaping was associated with 5.6% lower serum AMH compared to never-vapers (p=0.023), with no significant association observed for FSH. Current cigarette smoking was associated with a 9.4% reduction in AMH (p=0.005) and a 7.7% increase in FSH (p<0.001). Neither occasional nor former vaping or smoking was significantly associated with either marker. Sensitivity analyses excluding dual users of tobacco and e-cigarettes (n=64, 0.4% of sample) and excluding all former users produced consistent results. No significant interaction between smoking and vaping status was detected (p=0.271 for AMH; p=0.244 for FSH).

**Conclusions:**

In this large study of reproductively healthy women, current vape use was independently associated with lower AMH, a commonly used marker of ovarian reserve, after adjustment for smoking status and relevant confounders. These findings warrant further investigation, particularly given the widespread and growing use of e-cigarettes among women of reproductive age.

**Supplementary Information:**

The online version contains supplementary material available at https://doi.org/10.1186/s12905-026-04620-x.

## Introduction

Cigarette smoking is a global public health problem, with well documented health issues associated [1]. In terms of reproduction, smoking is well documented to be correlated with negative effects on fertility [2], ovarian reserve [3, 4], and has been associated with early onset menopause [5]. Furthermore, studies have shown reduced success with in vitro fertilisation (IVF) [6–8].

Vaping, or e-cigarette use, is considered by the Office for Health Improvement and Disparities to be a safer alternative to smoking, despite the lack of long-term studies [9]. The habit is rapidly growing in popularity, with an estimated one in five women of reproductive age (15–45 years old) now vaping, and numbers tripling since 2013 [10]. The chemical composition of the e-liquid within vapes in comparison to conventional cigarettes is hard to establish due to variations in models and methods of analysis, however, the effects of nicotine are said to be comparable. Moreover, other components, such as formaldehyde and metals, are reported to be present in equivalent, if not higher, levels [11]. Despite these concerns, very little research has been done to establish the impact of vaping on fertility.

Rodent studies have found that exposure to e-cigarette liquids significantly delays the time to first litter and reduces offspring numbers [12]. Similarly, mouse models have demonstrated increased follicle damage and impaired follicular development [13]. Whilst animal models have provided concerning early indications, the amount of human research is very limited. Initial studies have linked e-cigarette use to negative oocyte parameters in intracytoplasmic sperm injection cycles [3], however, no changes to oocyte number or maturity was observed in women seeking fertility treatment [14]. A larger population based study focusing on those actively trying to conceive showed a reduced fecundability rate [15]. These findings highlight the need for further research to fully establish the impact of vaping on fertility in women.

Anti-Müllerian hormone (AMH) is produced by granulosa cells of secondary, preantral, and early antral follicles and serum levels of AMH is commonly used as a marker of ovarian reserve [16]. Follicle stimulating hormone (FSH) has an indirect relationship with ovarian reserve and remains widely used as part of ovarian reserve assessment, particularly in the context of predicting ovarian response during IVF [16–18]. This study aimed to investigate the association between self-reported vaping, cigarette smoking, and hormonal markers of ovarian reserve, specifically AMH and FSH, in women of reproductive age in the United Kingdom. Based on the established literature on cigarette smoking, we hypothesised that current vaping may show a similar pattern of association, characterised by lower AMH and higher FSH. To our knowledge, this is the largest study to date examining the relationship between vaping behaviours and hormonal markers of ovarian reserve.

## Methods

### Study design and setting

This was a retrospective cross-sectional study using anonymised data collected from users of a private reproductive hormone testing service based in the UK between November 2022 and February 2025. Users accessed the service by completing an online health assessment (OHA) and subsequently returning an at-home capillary blood sample for hormonal analysis. Participants provided an at-home capillary blood sample, collected by finger-prick on day 3 of the menstrual cycle where applicable, for AMH and FSH analysis. Capillary blood testing has been shown to be comparable to venous sampling [19]. Blood samples were collected in Greiner MiniCollect^®^ CAT Serum Clot Activator Tube (Greiner Bio-One, Kremsmünster, Austria). All assays were performed by Inuvi Diagnostics Ltd (Gloucestershire, UK), a UKAS-accredited laboratory. Serum was separated by centrifugation as per the manufacturer’s instructions within three days of sample collection. As part of a larger panel of hormones, serum concentrations of AMH (pmol/L) were measured using the Access 2 Immunoassay System (Beckman Coulter, California, US). This assay has a measuring range of 0.14–171 pmol/L. The coefficient of variation (CV) for within-run repeatability is 2.6–4.5%. Follicle-stimulating hormone (FSH) (mIU/L) was measured using the Cobas^®^ e801 module (Roche Diagnostics, Basel, Switzerland). This assay has a measuring range of 0.3‑200 mIU/mL. The CV for repeatability and intermediate precision is 0.8–2.1% and 3.2–3.9%, respectively.

### Ethical approval and consent

This study involved the retrospective analysis of anonymised data collected from individuals who completed an OHA. At the time of participation, all individuals provided informed consent for their health and personal data to be used in anonymised form for research purposes. Consent was obtained electronically through an opt-in process embedded within the health assessment, with clear information provided regarding data usage, privacy, and confidentiality.

All data analysed were fully anonymised prior to access by the research team, and no identifiable information was used at any stage. The research protocol for the OHA and testing procedure was reviewed and approved by the London - Surrey Research Ethics Committee, which is part of the The National Health Service (NHS) Health Research Authority (HRA) (REC reference: 20/LO/0265).

All procedures were conducted in compliance with the ethical standards of the institutional and/or national research committee and in accordance with the 1964 Helsinki Declaration and its later amendments or comparable ethical standards.

### Participants

**Fig. 1:**
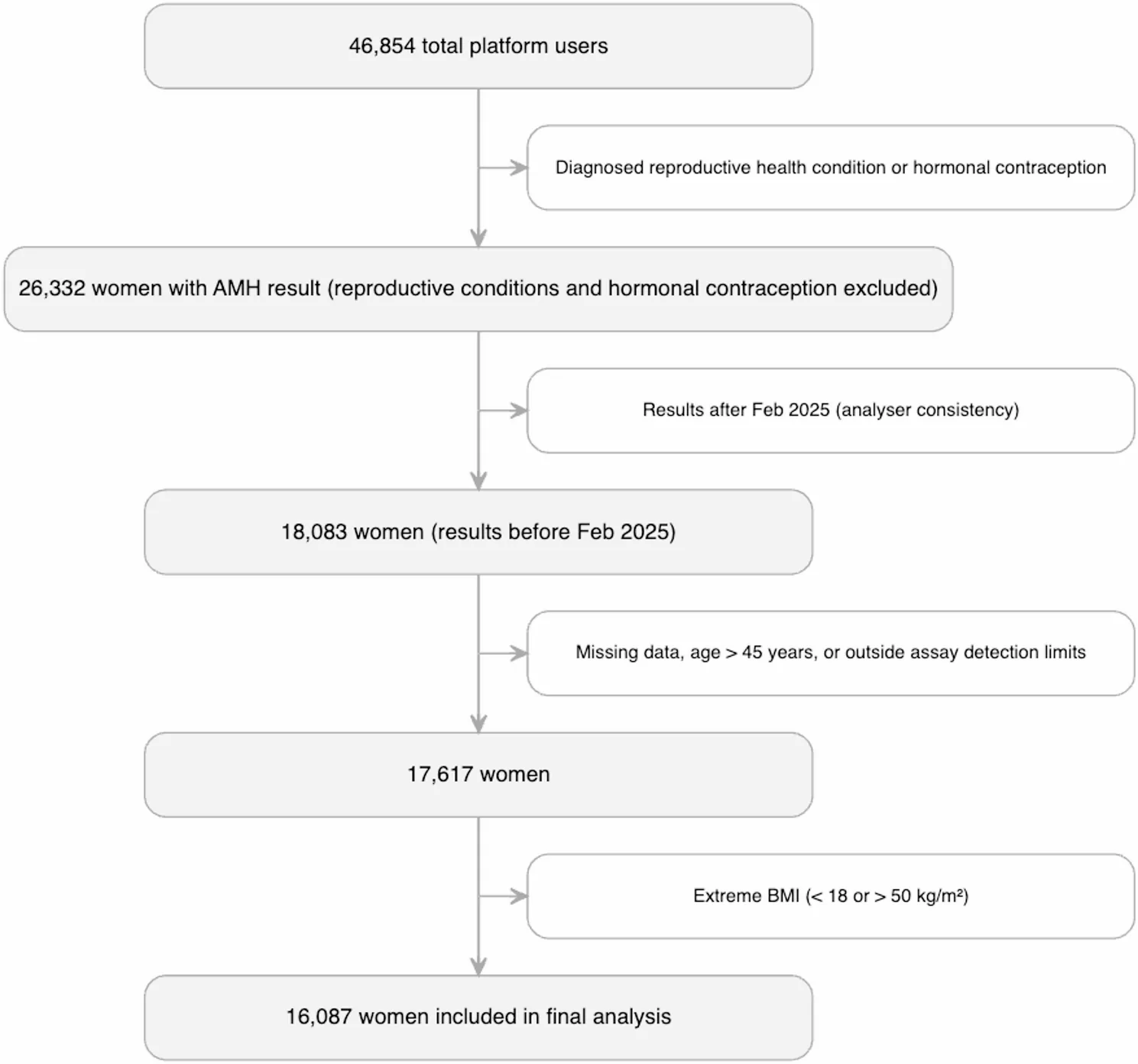
Flow diagram of participant numbers included in the study

Users aged 18–45 years were eligible for inclusion. Exclusion criteria were applied as follows: pre-existing reproductive health conditions including polyendocrine metabolic ovarian syndrome (PMOS) (previously polycystic ovary syndrome (PCOS)), hypothalamic amenorrhoea, relative energy deficiency in sport (RED-S), endometriosis, primary ovarian insufficiency (POI), and tubal blockage; blood results biochemically suggestive of PMOS or polycystic ovaries; current use of hormonal contraception; extreme or missing body mass index (BMI) values (below 18 or above 50 kg/m²); hormone values outside assay detection limits (< 0.05 pmol/L or > 200 pmol/L for AMH); and missing exposure or covariate data. After exclusion, 16,087 women were included in the final analysis (Fig. 1). The analytic sample was restricted to reports published prior to 15 February 2025 to ensure assay consistency, as the laboratory analyser used for AMH measurement was updated at this date. An additional 1,530 women were excluded following removal of extreme or anomalous BMI values. No formal a priori power calculation was performed because this was a retrospective analysis of all eligible available data. Findings are therefore interpreted using effect estimates and confidence intervals rather than post hoc claims of statistical power.

### Exposures and confounding variables

Vaping and smoking status was determined through the selection of one of four options in the OHA as “I have never vaped/smoked”, “I have quit vaping/smoking”, “Occasionally” or “Yes” and classified as never, former, occasional, or current use, respectively. However, no data on frequency nor duration were collected. Users self-reported their date of birth, weight, height, previous diagnoses of common reproductive health conditions, smoking and vaping habits, and use of contraception via their OHA. BMI was calculated by dividing the user’s weight (in kilograms) by their height (in metres squared). Ethnicity was based on 7 broad categories but due to small numbers condensed into White, Black, Asian, Mixed or Other (which includes Middle Eastern, Indigenous/First nations and any others not listed).

### Statistical analysis

Descriptive statistics (frequency, percentage, mean, standard deviation (SD)) were calculated for all demographic variables and for AMH and FSH by exposure group. Multiple linear regression was used to assess the associations between e-cigarette and cigarette smoking with each ovarian reserve marker. Due to non-normal distribution of hormone values and improved model fit, both AMH and FSH were log-transformed prior to analysis. Age, BMI, and ethnicity were included as confounding variables. Age has been demonstrated in several studies to have a modulating effect on AMH, after the age of 25 a progressive decline is seen with increasing age [17]. Due to the non-linear association between age and AMH the model also included quadratic age. This was determined through comparison of models using linear, polynomial and spline regression models with quadratic showing the greatest R2 valued and lowest AIC and agrees with several previous studies looking at AMH and age [20, 21]. To evaluate whether a quadratic age term was similarly warranted for FSH, models with linear and quadratic age were compared using the likelihood ratio test and AIC; the quadratic specification was selected for the FSH model (quadratic AIC 12,222 vs. linear AIC 12,410; *p* < 2 × 10⁻¹⁶). Ethnicity has been associated with substantial variations in AMH and a suggested negative effect of BMI on AMH, particularly in those who are obese has been demonstrated [22, 23]. An ethnic-specific variation in the pituitary-ovarian relationship has been suggested, altering FSH levels based on ethnicity and BMI has been negatively correlated with FSH [24, 25]. Percentage change in AMH and FSH were estimated from the coefficient generated from the regression model using the following equation: (exp(coefficient) − 1)*100. The final model included both vaping, smoking and all confounding variables so accounted for overlap between the two behaviours. To assess the robustness of the primary findings, three pre-specified sensitivity analyses were conducted: (i) exclusion of women reporting concurrent current vaping and current smoking (dual users; *n* = 64); (ii) exclusion of all former users of either behaviour, retaining only current and never users; and (iii) stratification of the primary vaping-AMH association by age group (under 30, 31–35, over 35 years). A formal test for multiplicative interaction between vaping and smoking status was conducted using an F-test comparing the additive main-effects model to a model including the vaping × smoking interaction term. Collinearity among predictors was assessed using the generalised variance inflation factor (GVIF). Given the hypothesis-driven nature of the primary analyses, no formal correction for multiple comparisons was applied; p-values for secondary and sensitivity analyses were interpreted cautiously. All analyses were conducted using R studio Version 2025.05.0 + 496 (2025.05.0 + 496).

## Results

### Participants

16,087 women aged 18–45 years were included in the final analysis (Fig. 1). Of the whole cohort the mean AMH value was 16.3 pmol/L (SD = 9.44) and the mean FSH value was 8.21 IU/L (SD = 4.43). Demographic information is presented in Table 1. Within this cohort, current vaping was more prevalent than current smoking, with 1,023 (6.4%) women reporting current vaping and 437 (2.7%) reporting current smoking. Overall, 64 women (0.4% of the sample; 6.3% of current vapers; 14.6% of current smokers) reported concurrent current use of both tobacco and e-cigarettes. The distribution of vaping status varied by age band. Current vaping was proportionally highest among women aged ≤ 20 years (16.3%), although this was the smallest age stratum (*n* = 43), followed by women aged 21–25 years (10.4%). The proportion reporting current vaping then decreased across older age groups, from 6.7% among women aged 26–30 years to 4.9% among women aged 41–45 years. Current smoking was less clearly age-patterned, ranging from 2.3% among women aged ≤ 20 years to 4.5% among women aged 21–25 years and 2.9% among women aged 41–45 years. Full age-stratified distributions of vaping and cigarette smoking status are provided in Supplementary Tables S1 and S2, respectively.

**Table 1:** Frequency and percentage of demographic data from 16,087 women in the cohort, including mean and standard deviation (SD) for age and BMI and for each stratification mean and standard deviation of AMH pmol/L and FSH IU/L

		Frequency	Proportion	AMH pmol/L mean (SD)	FSH IU/L mean (SD)
Age	≤ 20	43	0.30%	23.14 (7.6)	6.72 (1.7)
Mean (SD) = 32.4 (4.5)	21–25	763	4.70%	21.61 (8.8)	7.14 (3.1)
	26–30	4663	29%	19.53 (9.2)	7.36 (2.8)
	31–35	7231	44.90%	17.16 (9)	7.93 (3.2)
	36–40	2585	16.10%	9.74 (5.9)	9.42 (5.8)
	41–45	802	5%	5.33 (4.1)	12.76 (10.3)
BMI	Underweight	248	1.50%	18.01 (8.8)	8.55 (5)
Mean (SD) = 25.8 (5.8)	Normal	8657	53.80%	16.6 (9.5)	8.33 (4.7)
	Overweight	4005	24.90%	16.35 (9.6)	8.03 (3.9)
	Obese	3177	19.70%	15.25 (9.1)	8.05 (4.2)
Ethnicity	Asian	975	6.10%	16.18 (9.3)	8.21 (4.4)
	Black	411	2.60%	16.18 (11.3)	9.12 (7.7)
	Mixed	705	4.40%	16.83 (9.5)	7.87 (3.1)
	White	13,264	82.50%	16.28 (9.4)	8.19 (4.4)
	Other	732	4.60%	16.13 (10)	8.32 (4.1)
Vaping	I have never vaped	12,404	77.10%	16.1 (9.4)	8.27 (4.5)
	I have quit vaping	1537	9.60%	17.02 (9.1)	7.98 (4.6)
	Occasionally	1123	7%	17.53 (9.8)	7.98 (3.7)
	Yes	1023	6.40%	16.09 (9.6)	8.06 (4.4)
Smoking	I have never smoked	10,842	67.40%	16.55 (9.4)	8.12 (4.2)
	I have quit smoking	3525	21.90%	15.37 (9.3)	8.44 (5)
	Occasionally	1283	8%	16.96 (9.3)	7.99 (3.5)
	Yes	437	2.70%	15.23 (10.1)	9.03 (6.8)

### AMH

**Fig. 2:**
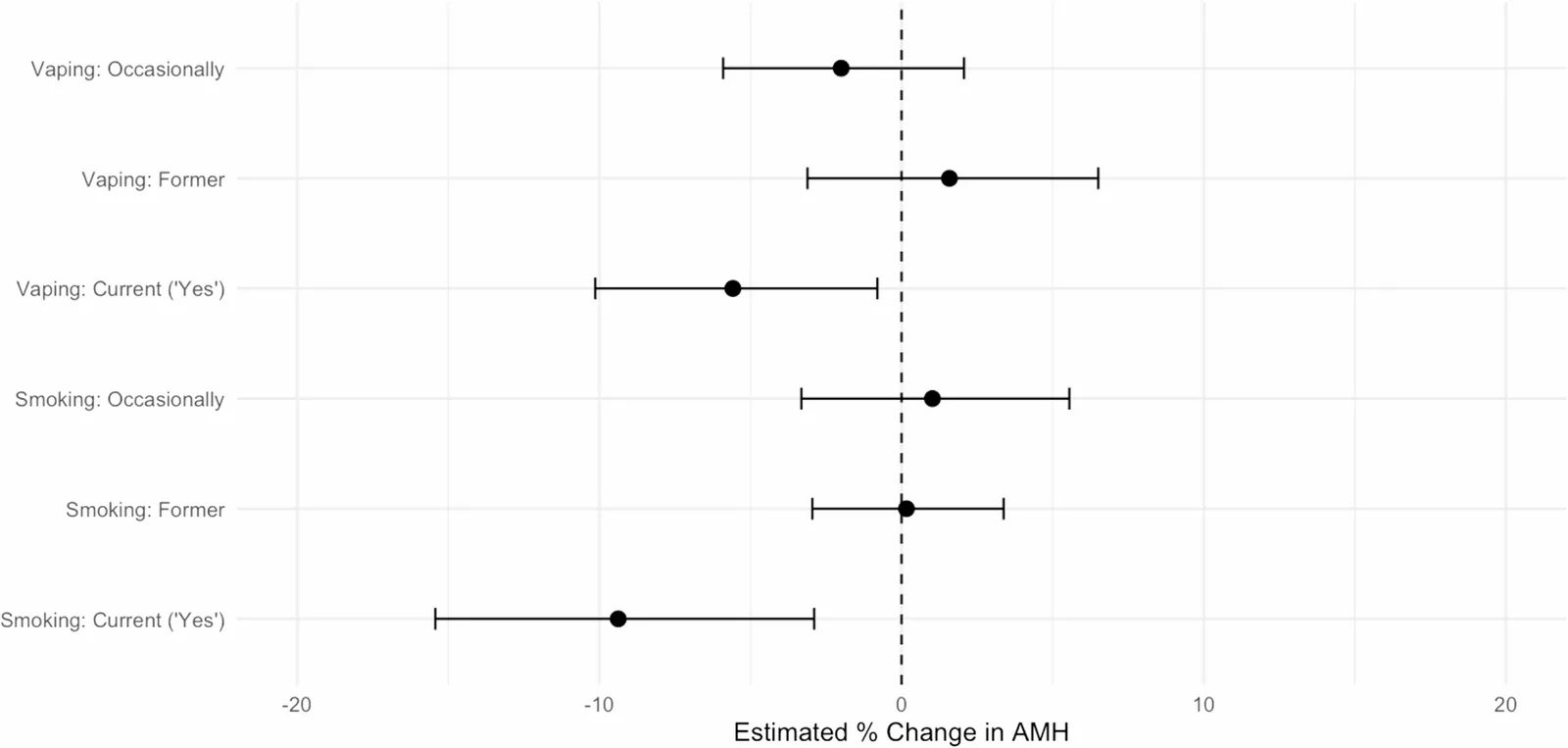
Association of vaping and smoking habits with serum AMH in 16,087 women aged 18–45 years. Estimated percentage difference in AMH levels with 95% confidence intervals (CIs) from the multivariable linear regression model, adjusted for age (linear + quadratic), BMI, and ethnicity. The reference categories are “I have never vaped” and “I have never smoked.” The dashed line indicates no difference (0%). CIs crossing the dashed line indicate non-significant associations. Current vaping was associated with 5.6% lower AMH (95% CI − 10.1% to − 0.8%, *p* = 0.023) and current smoking was associated with 9.4% lower AMH (95% CI − 15.4% to − 2.9%, *p* = 0.005) compared to the respective never-use reference categories

Multiple linear regression was used to assess the relationship between vaping and AMH, adjusted for confounding variables (Fig. 2). Women who reported current vaping (“Yes”) had significantly lower AMH levels, corresponding to a 5.6% decrease compared to those who had never vaped (β = −0.0574, 95% CI -10.1% to -0.8%, *p* = 0.023). Current smoking (“Yes”) was also significantly associated with lower AMH, at 9.4% lower than never-smokers (β = -0.0985, 95% CI -15.4% to -2.9%, *p* = 0.005). Neither occasional vaping (-2.0%, 95% CI -5.9% to 2.1%, *p* = 0.331) nor former vaping (+ 1.6%, 95% CI -3.1% to 6.5%, *p* = 0.515) was significantly associated with AMH. Neither occasional nor former smoking was significantly associated with AMH. No significant interaction between vaping and smoking status was detected for AMH (F-test, *p* = 0.271), supporting the use of an additive main-effects model. Collinearity among predictors was low, with generalised VIF values below 1.15 for all substantive predictors (vaping GVIF-equivalent = 1.14; smoking GVIF-equivalent = 1.15); the elevated VIF for the age polynomial terms (≈ 106) reflects expected mathematical collinearity between age and age² and does not affect other coefficient estimates.

### FSH

**Fig. 3:**
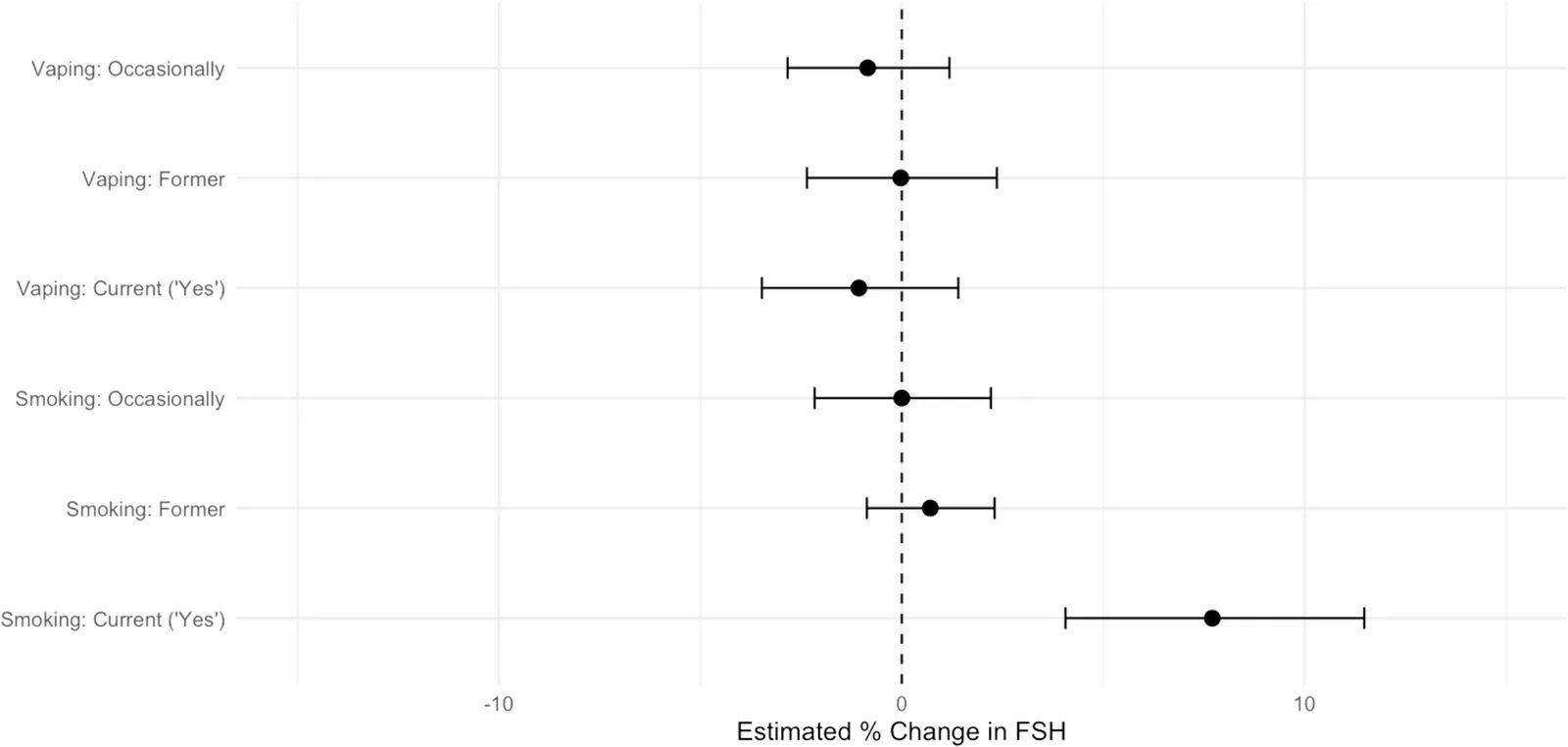
Association of vaping and smoking habits with serum FSH in 16,087 women aged 18–45 years. Estimated percentage difference in FSH levels with 95% CIs from the multivariable linear regression model, adjusted for age (linear + quadratic), BMI, and ethnicity. The reference categories are “I have never vaped” and “I have never smoked.” The dashed line indicates no difference (0%). Current smoking (“Yes”) was associated with a 7.7% increase in FSH (95% CI 4.1% to 11.5%, *p* < 0.001) compared to never-smokers

No significant association was observed between any vaping category and FSH levels (current vaping: -1.1%, 95% CI -3.5% to 1.4%, *p* = 0.394). Current cigarette smoking (“Yes”) was associated with a 7.7% increase in FSH (β = 0.0742, 95% CI 4.1% to 11.5%, *p* < 0.001). Neither occasional nor former smoking was significantly associated with FSH. Model selection for FSH favoured inclusion of a quadratic age term over a linear age specification (quadratic AIC = 12,222 vs. linear AIC = 12,410; LR test *p* < 2 × 10⁻¹⁶). No significant interaction between vaping and smoking was detected for FSH (F-test, *p* = 0.244) (Fig. 3).

### Sensitivity analyses

To verify that the association between current vaping and AMH was not attributable to concurrent tobacco smoking, we excluded 64 women (0.4% of the analytic sample) who reported simultaneous current vaping and current smoking. Effect estimates were unchanged: current vaping remained independently associated with a 5.9% reduction in AMH (95% CI -10.6% to -1.0%, *p* = 0.019) and current smoking with a 10.1% reduction (95% CI -16.5% to -3.2%, *p* = 0.005). The association between current smoking and FSH was similarly robust to dual-user exclusion (+ 7.5%, 95% CI 3.6% to 11.5%, *p* < 0.001). A second sensitivity analysis restricted to current and never users only (excluding all former users; *n* = 12,169) yielded consistent directional findings for current smoking on AMH (-9.7%, 95% CI -16.1% to -2.8%, *p* = 0.007) and FSH (+ 7.1%, 95% CI 3.3% to 11.1%, *p* < 0.001). The association between current vaping and AMH was attenuated and no longer statistically significant in this restricted sample (-3.1%, 95% CI -10.4% to 4.9%, *p* = 0.436). This may reflect reduced precision after excluding former users, but it also indicates that the vaping-AMH association should be interpreted cautiously. The direction of effect remained consistent with the main analysis, but the loss of statistical significance underscores the need for replication in prospective cohorts with more detailed exposure characterisation. Age-stratified analyses of the vaping-AMH association across three strata (under 30 years, *n* = 5,466; 31–35 years, *n* = 7,231; over 35 years, *n* = 3,387) showed consistent negative directional associations between current vaping and AMH in all strata, with estimated reductions of 6.2% (under 30, *p* = 0.069), 4.1% (31–35, *p* = 0.267), and 7.4% (over 35, *p* = 0.270). None reached statistical significance within individual strata, reflecting reduced power compared to the full-sample analysis (Supplementary Table S3). A summary of sensitivity analysis results for the primary exposures (current vaping and current smoking) is presented in Table 2.

**Table 2:** Sensitivity analysis results for current vaping and current smoking. Estimated percentage change in AMH and FSH (with 95% CIs and p-values) from the main model, a model excluding dual users of tobacco and e-cigarettes (*n* = 64 excluded), and a model restricted to current and never users (former users excluded; *n* = 12,169). Bold *p*-values indicate statistically significant associations (*p* < 0.05)

Outcome	Model	Exposure	% Change	95% CI	*p*-value
AMH	Main model (*n* = 16,087)	Current vaping	-5.60%	-10.1% to -0.8%	**0.02276**
		Current smoking	-9.40%	-15.4% to -2.9%	**0.00524**
	No dual users (*n* = 16,023)	Current vaping	-5.90%	-10.6% to -1%	**0.01876**
		Current smoking	-10.10%	-16.5% to -3.2%	**0.00501**
	No former users (*n* = 12,169)	Current vaping	-3.10%	-10.4% to 4.9%	0.4359
		Current smoking	-9.70%	-16.1% to -2.8%	**0.00656**
FSH	Main model (*n* = 16,087)	Current vaping	-1.10%	-3.5% to 1.4%	0.394
		Current smoking	7.70%	4.1% to 11.5%	**< 0.001**
	No dual users (*n* = 16,023)	Current vaping	-1.20%	-3.6% to 1.4%	0.365
		Current smoking	7.50%	3.6% to 11.5%	**< 0.001**
	No former users (*n* = 12,169)	Current vaping	-1.20%	-5% to 2.8%	0.546
		Current smoking	7.10%	3.3% to 11.1%	**< 0.001**

## Discussion

This large cross-sectional study of 16,087 reproductively healthy women in the UK found that current e-cigarette use was independently associated with a 5.6% lower serum AMH after adjustment for age, BMI, ethnicity, and cigarette smoking status. No significant association was observed between e-cigarette use and FSH. Current cigarette smoking was associated with both reduced AMH (− 9.4%) and elevated FSH (+ 7.7%), consistent with the established literature. To our knowledge, this is the largest study to date to investigate the association between e-cigarette use and hormonal markers of ovarian reserve in a population of reproductively healthy women.

No published studies have examined the association of vaping with serum levels of FSH and AMH. Although the observed 5.6% lower AMH among current vapers was statistically significant at the population level, the absolute difference was small, approximately 0.71 pmol/L. Given known biological and analytical variability in AMH measurement, this difference should not be interpreted as evidence of clinically meaningful ovarian reserve reduction in an individual woman. Instead, it may represent a modest population-level association and an early signal that requires confirmation in prospective studies. The association may nonetheless be representative of a subtle but real impact on follicular pool and thus ovarian reserve [26]. Only one study has looked at AMH in follicular fluid in e-cigarette users and no significant changes were found [14]. However, the vaping cohort in the study was small, consisting only of 42 people, and reported very low nicotine exposure levels from estimated usage (based on product type and usage). Another survey of North American women trying to conceive found a small reduction in fecundability for those who currently smoked e-cigarettes [15]. Lastly, evidence from a mouse study has indicated impaired follicular development following exposure to vape fumes [13]. Collectively, these suggest a potential harmful association between vaping and fertility. Our study adds to this body of data showing evidence of lower ovarian reserve markers for current vapers.

Current smoking was found to have a negative impact on markers of ovarian reserve, significantly affecting both AMH and FSH levels. These findings are consistent across sensitivity analyses excluding dual users and former users, supporting the robustness of the associations. This is unsurprising as smoking has been well documented to have a negative effect on ovarian reserve markers, and several studies have seen a similar significant lower serum AMH [3, 4, 27–30]. In our study neither occasional nor past smoking were found to have a detrimental effect on AMH or FSH levels. This aligns with other studies finding past smoking to have no effect on AMH [29, 31] or FSH [31]. Reduced AMH levels have also been found in the follicular fluid of women who smoke [32]. Freour et al. observed fewer small follicles (2–5 mm) in smoking women, based on antral follicle counts (AFC) [27], potentially explaining the reduced serum AMH observed. Elevated FSH is indicative of reduced ovarian reserve, as the amount of FSH produced increases due to a lack of responsiveness from the ovaries [16]. Several others have also found increased FSH in smokers [33–35], including Windham et al. [36], who found this to be particularly prevalent in heavy smokers.

Not all studies have found smoking to be associated with lower markers of ovarian reserve, with some reporting no change in AMH levels [31, 37–40]. The majority of studies investigating the association between ovarian reserve and smoking habits thus far have been conducted in infertile populations, however, research conducted within the general population [37, 38] or in those with positive reproductive history [31, 41] were the ones who found no significant associations. This study represents the general UK population including women not currently attempting to conceive, which is an important distinction from most prior work conducted in infertility clinic populations. Selection into infertility treatment introduces confounding by indication and may distort associations with ovarian reserve. Discrepancies between studies may arise due to sample sizes varying greatly. The previously mentioned studies only had between 30 and 83 smokers, with the exception of Hawkins-Bressler et al. which had 318, which are much smaller compared to over 1800 active or occasional smokers in the current study. Dólleman et al. [4] is the largest study so far to investigate smoking and AMH within the general population and they agreed with our findings seeing a reduced AMH in those who smoked, suggesting the other studies may be underpowered. Some studies that found no association with AMH, did find associations with other markers indicative of a decreased ovarian reserve. Two found smoking increased FSH [31, 33] and another observed a decreased AFC [42], despite no significant changes in AMH.

Several biological mechanisms have been proposed for the observed associations between smoking, vaping and ovarian reserve markers, although most evidence remains indirect, experimental, or derived from smoking rather than vaping-specific human studies. Nicotine has been demonstrated to affect follicular growth, death and normal steroidogenesis [43]. Several studies have confirmed the presence of cotinine (the major nicotine metabolite) in follicular fluid to be significantly higher in women who smoke [14, 32, 44]. A strong positive correlation between the average number of cigarettes smoked per day and cotinine levels within follicular fluid has also been found [45]. Trapphoff et al. (2024) identified 6 times the levels of cotinine in follicular fluid in those who vaped, despite low reported nicotine intake, however, this was not significantly different when compared to those who did not smoke or vape [14]. There is evidence vaping may increase nicotine exposure. In a study examining ad-hoc vaping over a 90-minute period, vapers were found to intake an amount of nicotine equivalent to 3–4 cigarettes, with a steady increase in nicotine levels peaking at the end of study, unlike a bolus delivery of nicotine delivered through the consumption of a single cigarette [46].

However, nicotine may not be the only causative agent. Multiple ingredients found in cigarettes, such as Benzo[a]pyrene (BaP), alkaloids, and cadmium, have been linked to mechanisms that diminish ovarian reserve [43, 47, 48]. Studies have detected the presence of heavy metals including cadmium and polycyclic hydrocarbons like BaP, in e-cigarettes as well [11]. Moreover, the vapour generated from e-cigarettes contains a mixture of diverse chemicals including formaldehyde, which has demonstrated negative effects on follicle number and maturity in rats and volatile organic compounds which adversely affect endocrine functions [11]. Further investigations into flavouring liquids have shown that some have cytotoxic effects independent of nicotine content, and large variations in potency between brands and flavours [49]. The huge variation in flavouring chemicals are often not included on product labelling and have no safety guarantee, particularly over long-term use [50]. Unfortunately, the composition of vapes and e-cigarettes varies greatly between manufacturer and model, making it difficult to assess chemical exposure and compare to traditional cigarettes [11].

Our study did not collect duration of years smoking or vaping, however, previous studies have identified negative associations with duration of smoking and AMH levels [41, 51], and increased odds of earlier age at menopause [5]. In caucasian women, older age was associated with an increased number of years smoking [33]. IIn our study, current vaping was more prevalent in younger age groups and generally decreased with increasing age. Jackson et al. [10] found the prevalence of vaping has tripled since 2013, but most of this increase occurred after 2020. As the prevalence of vaping has only taken off in recent years, the long-term reproductive associations of extended and continued use may not yet be clear. Further work on specific vaping behaviours, including duration of vaping and quantification on vape use, exposure to nicotine, and other chemicals, and their relationship with fertility markers and reproductive outcomes is essential.

### Strengths and limitations

Although the cohort included over 16,000 women and had ethnic representation broadly reflective of UK census data [52], the fee-paying and self-selected nature of the service limits generalisability to the wider UK population.

Limitations include the cross-sectional analysis and use of serum ovarian reserve markers only. Further analysis incorporating AFC would allow a more clinically complete assessment of the relationship between vaping and ovarian reserve markers. Both AMH and FSH can vary between cycles, which might affect the interpretation of our results. However, although we did not test multiple times and could not correct for intercycle variability, we had the advantage of using the same assay across the cohort and all participants tested on day 3 of their cycle, thus reducing the intracycle variability [52]. The cross-sectional design and absence of biochemical exposure validation (e.g. urinary cotinine) are important limitations. Self-reported smoking and vaping status could not be quantified in terms of frequency, duration, nicotine concentration, or device type, precluding dose-response analysis. This is a significant limitation as misclassification of exposure is possible, and results are likely biased towards the null. Several potentially relevant confounders were not available in the dataset, including alcohol consumption, cannabis use, educational attainment, and chronic psychological stress, all of which may independently influence ovarian reserve and correlate with smoking or vaping behaviours. Other unmeasured factors, including reproductive history, menstrual regularity, occupational or environmental exposures and broader lifestyle behaviours, may also have contributed to residual confounding. The service is accessed on a fee-paying basis and therefore likely over-represents women of middle to higher socioeconomic status. Given that e-cigarette use is disproportionately prevalent in lower socioeconomic groups in the UK [53], this discrepancy may introduce socioeconomic selection bias. The direction and magnitude of this bias are difficult to determine, although under-representation of lower socioeconomic groups may have attenuated observed associations if exposure prevalence and cumulative exposure were lower in this cohort than in the wider population. The study did not include AFC or clinical reproductive outcomes, which limits interpretation in a fully clinically meaningful sense. Further detail on vaping and smoking behaviours including duration, amount and estimated exposures would allow for a more comprehensive analysis and greater understanding into this relationship. Self-reported data allows for the possibility of social desirability bias and underreporting of negatively perceived habits. This is reduced due to the anonymity of using an online assessment. Further to this, recall bias may occur due to misunderstanding or erroneously recalling past experiences. The cohort may be biased to those of a higher socio-economic status due to the initial cost of the test. Any public health implications from these findings must be considered conditional upon replication in prospective longitudinal studies incorporating objective exposure measures, including urinary cotinine measurement, device-type characterisation, nicotine concentration, and duration of use.

### Implications for public health

While this study does not include longitudinal fertility outcomes such as time to pregnancy or live birth rates, the use of validated biomarkers provides non-invasive indicators of ovarian reserve. Importantly, this cohort includes women not currently attempting to conceive and is not restricted to infertility clinic patients, overcoming limitations of previous studies where results may be influenced by selection bias or infertility-specific confounding factors. Our findings may inform future public health messaging efforts, particularly if confirmed by longitudinal research. Given that vaping is often perceived as a safer alternative to smoking, emerging evidence suggesting biological associations with ovarian reserve markers warrants greater attention. Should future prospective research with objective exposure characterisation substantiate a causal relationship between vaping and diminished ovarian reserve, this would provide stronger justification for policy updates to include reproductive health warnings in anti-vaping guidance. We caution against direct policy inference from cross-sectional, self-reported exposure data alone.

### Future research

In 2024, in the UK, 11% of the adult population was vaping, which is higher than the population of smokers (11.9%) and 18% of 11-17-year-olds had tried vaping [54], highlighting the scale of potential reproductive health exposure in current and future cohorts of women of reproductive age. Future research should prioritise large prospective cohort studies with propensity score matching, incorporating detailed exposure characterisation including frequency, device type, nicotine concentration, and duration of use, alongside biological validation through urinary cotinine measurement. Future studies should also incorporate AFC and repeated AMH measurement to better distinguish biological variation from persistent differences in ovarian reserve markers. Crucially, studies must move beyond ovarian reserve markers to examine whether e-cigarette use affects follicular quality and natural conception rates.

## Conclusions

This large, UK-based cross-sectional study found that current e-cigarette use was associated with 5.6% lower serum AMH after adjustment for age, BMI, ethnicity, and cigarette smoking status. No significant association between vaping and FSH was observed. Current cigarette smoking was associated with both reduced AMH (-9.4%) and elevated FSH (+ 7.7%), consistent with the established literature. These associations were robust to sensitivity analyses excluding concurrent tobacco and e-cigarette users and excluding former users. While the absolute magnitude of the vaping-AMH association falls below the threshold of individual clinical significance, our findings represent the largest community-based analysis to date of an association between e-cigarette use and a commonly-used marker of ovarian reserve. Results should be interpreted with caution given the cross-sectional design, reliance on self-reported exposure, and absence of biochemical validation. Longitudinal studies with objective exposure characterisation are needed to establish causality and determine the reproductive health implications of long-term vaping.

## Supplementary Information

Supplementary Material 1.

## Data Availability

The data analysed during this study are not publicly available owing to commercial confidentiality and data protection obligations of the service provider. De-identified data may be made available to the editors of the journal pre- and/or post-publication for review or query upon reasonable request to the corresponding author.

## Abbreviations

AMH: Anti-Müllerian hormone
FSH: Follicle-stimulating hormone
BMI: Body mass index
CI: Confidence interval
GVIF: Generalised variance inflation factor
OHA: Online health assessment
CV: Coefficient of variation
AFC: Antral follicle count
IVF: In vitro fertilization
PMOS: Polyendocrine metabolic ovarian syndrome
POI: Primary ovarian insufficiency
UK: United Kingdom
TSH: Thyroid-stimulating hormone
VIF: Variance inflation factor
