# Supplementary material for: E-cigarette use (vaping) and cigarette smoking are associated with reduced markers of ovarian reserve: a cross-sectional study of 16,087 women
Source: BMC Womens Health. 2026 Jun 23;26:437. doi: 10.1186/s12905-026-04620-x (PMC13543381; doi:10.1186/s12905-026-04620-x)
Supplement: Supplementary file 1 [file 12905_2026_4620_MOESM1_ESM.pdf]

Age stratum	Never vaped n (%)	Former vaping n (%)	Occasional vaping n (%)	Current vaping n (%)	Total
≤20	25 (58.1%)	6 (14%)	5 (11.6%)	7 (16.3%)	43
21-25	470 (61.6%)	93 (12.2%)	121 (15.9%)	79 (10.4%)	763
26-30	3437 (73.7%)	385 (8.3%)	529 (11.3%)	312 (6.7%)	4663
31-35	5646 (78.1%)	478 (6.6%)	676 (9.3%)	431 (6%)	7231
36-40	2140 (82.8%)	128 (5%)	162 (6.3%)	155 (6%)	2585
41-45	686 (85.5%)	33 (4.1%)	44 (5.5%)	39 (4.9%)	802

Supplementary Table S1. *Distribution of vaping status by age stratum. Values are presented as n (%) within each age stratum. Percentages are calculated within age strata and therefore sum to 100% across vaping categories. This table provides the age-specific distribution of never, former, occasional and current vaping status to support assessment of age-related differences in exposure distribution and potential residual confounding.*

Age stratum	Never smoked n (%)	Former smoking n (%)	Occasional smoking n (%)	Current smoking n (%)	Total
≤20	27 (62.8%)	4 (9.3%)	11 (25.6%)	1 (2.3%)	43
21-25	489 (64.1%)	135 (17.7%)	105 (13.8%)	34 (4.5%)	763
26-30	3289 (70.5%)	828 (17.8%)	431 (9.2%)	115 (2.5%)	4663
31-35	4941 (68.3%)	1611 (22.3%)	503 (7%)	176 (2.4%)	7231
36-40	1641 (63.5%)	680 (26.3%)	176 (6.8%)	88 (3.4%)	2585
41-45	455 (56.7%)	267 (33.3%)	57 (7.1%)	23 (2.9%)	802

Supplementary Table S2. *Distribution of cigarette smoking status by age stratum. Values are presented as n (%) within each age stratum. Percentages are calculated within age strata and therefore sum to 100% across smoking categories. This table provides the age-specific distribution of never, former, occasional and current cigarette smoking status to support assessment of age-related differences in exposure distribution and potential residual confounding.*

Age stratum	Exposure	% Change	95% CI	p-value
AMH (Under 30)	Occasional vaping	-0.40%	-5.6% to 5.2%	0.893
AMH (Under 30)	Former vaping	2.90%	-3.3% to 9.6%	0.367
AMH (Under 30)	Current vaping	-6.20%	-12.4% to 0.5%	0.069
AMH (31-35)	Occasional vaping	-2.50%	-8.1% to 3.4%	0.3945
AMH (31-35)	Former vaping	2.30%	-4.7% to 9.8%	0.5292
AMH (31-35)	Current vaping	-4.10%	-10.9% to 3.3%	0.2667
AMH (Over 35)	Occasional vaping	-4.70%	-16.5% to 8.8%	0.476
AMH (Over 35)	Former vaping	-2.80%	-16.2% to 12.8%	0.71
AMH (Over 35)	Current vaping	-7.40%	-19.3% to 6.2%	0.27

Supplementary Table S3. *Age-stratified associations between vaping status and serum AMH across three age strata (under 30 years, 31–35 years, and over 35 years). Results are from multivariable linear regression models fitted separately within each stratum, adjusted for age (linear and quadratic terms), BMI, ethnicity, and smoking status. The reference category for all comparisons is "I have never vaped." Percentage change and 95% CIs were derived from model coefficients using the formula (exp(β) − 1) × 100. None of the within-stratum associations reached statistical significance, reflecting reduced statistical power compared to the full analytic sample (n = 16,087). AMH: anti-Müllerian hormone.*
